# Experimental Study and Modelling of Fresh Behaviours of Basalt Fibre-Reinforced Mortar Based on Average Water Film Thickness and Fibre Factor

**DOI:** 10.3390/ma16062137

**Published:** 2023-03-07

**Authors:** Leo Gu Li, Pui-Lam Ng, Kai-Long Zeng, Hui-Zhu Xie, Cong-Mi Cheng, Albert Kwok-Hung Kwan

**Affiliations:** 1 School of Civil Engineering, Guangzhou University, Guangzhou 510006, China; 2 Department of Civil Engineering, The University of Hong Kong, Hong Kong 999077, China; 3 Faculty of Civil Engineering, Vilnius Gediminas Technical University, LT-10223 Vilnius, Lithuania; 4 China Jinmao Holdings Group Limited, Guangzhou 510000, China; 5 School of Civil and Transportation Engineering, Guangdong University of Technology, Guangzhou 510006, China

**Keywords:** average water film thickness, basalt fibre, fibre factor, fibre mortar, fresh behaviour

## Abstract

Though previous studies have indicated that the fresh behaviours of plain mortar/concrete are mainly governed by the average water film thickness (AWFT), whether the concept of AWFT is also applicable to fibrous mortar/concrete still needs to be explored. Furthermore, for fibrous mortar/concrete, it is obvious that the fibres added also have certain effects on the fresh behaviours. In two previous studies on basalt fibre-reinforced mortar (BFRM), the integral effects of the AWFT and fibre dosage as well as the integral effects of the AWFT and fibre length were individually investigated. In this study, a fibre factor (FF) defined as the fibre volume multiplied by the fibre aspect ratio was employed and 24 extra mortar groups were tested. A total of 68 mortar groups were applied in numerical analysis. The results of the regression analysis yielded good correlations of the workability, fluidity, cohesiveness, and adhesiveness of BFRM with the AWFT and FF, suggesting that the AWFT and FF are together the governing parameters controlling the fresh behaviours of BFRM. Hence, the AWFT and FF may be used to develop a model for the fresh properties of BFRM.

## 1. Introduction

As the second widely used material in construction only after water, cement-based material (CBM), or mortar/concrete, has many advantages [1,2]. One major advantage is mouldability, which allows the CBM to be formed into different shapes [3,4]. The mouldability is highly dependent on the fresh behaviours of the CBM, and the key parameters affecting the fresh behaviours are still being explored. Early in the 1960s, Powers [5] put forward the excess paste theory and pointed out that it should be the excess paste (in excess of the amount needed to fill up the voids between aggregate particles) that makes the CBM flowable. Later, in the 1980s, Helmuth [6] postulated that the fresh behaviours of CBM are governed by the thickness of the water film coating the solid grains. Since then, numerous studies have been launched along this line. Denoting the average water film thickness (AWFT) as the ratio of excess water (in excess of the amount needed to fill up the voids between solid particles) to solid surface area, it has been proven that the AWFT is the major parameter affecting the fresh behaviours of plain CBM without fibres [7,8,9,10,11,12,13].

For a fibre-reinforced cement-based material (FRCBM), or fibrous mortar/concrete, in addition to the concrete mix parameters [14,15,16], the fibres added also have certain influences on the fresh behaviours [17,18,19]. Soroushian and Bayasi [20] revealed that the shape of the steel fibres exhibits great influence on the fluidity of fibrous concrete mixes. Jiao et al. [21] observed that a higher glass fibre content would increase the shear-thickening intensity of the mortar. Gültekin et al. [22] reported that the increase in length of basalt fibre would reduce the fluidity of self-consolidating concrete (SCC). Hossain et al. [23] pointed out that adding polyvinyl alcohol fibres can significantly improve the cohesiveness of SCC. Overall, the influences of the different fibre parameters on the rheological behaviours appear to be quite complicated and up to now are not fully understood.

Notwithstanding that it is a difficult task to theoretically model the fibre effects on the fresh behaviours of FRCBM, some sound experimental explorations have been carried out. For instance, Hannant [24] pointed out that the fibre volume (as a ratio of FRCBM volume) and the fibre aspect ratio (ratio of the fibre length to fibre diameter) are two main parameters influencing the fresh and hardened performance of FRCBM. Hughes and Fattuhi [17] put forward a fibre factor of V^1/2^ (L/D), in which V is the fibre volume, L is the fibre length, and D is the fibre diameter, and showed that this factor has a dominant effect on the workability of steel fibrous concrete. Groth [25] applied a fibre factor of V (L/D) to carry out a mix design of fibre-reinforced SCC. Grünewald [26] used the fibre factor of V (L/D) and the “relative water layer thickness” parameter to build up a performance-based mix design method for fibrous SCC. Recently, the authors’ research group [18] found that both the AWFT and fibre factor of V (L/D) play important roles in the fluidity of polypropylene fibre-reinforced mortar.

Regarding basalt fibre-reinforced mortar (BFRM), the authors’ research group had previously launched two separate studies on investigating the combined effects of AWFT and fibre dosage [27] as well as the combined effects of AWFT and fibre length [28] on the fresh behaviours. The results revealed that AWFT plays the dominant role in the fresh behaviours, whereas the fibre dosage and fibre length both exert important influences. However, due to the limited scopes of these previous studies, the possibility of developing a fibre factor (FF) specifically for BFRM and the role of such FF in the fresh behaviours of BFRM have not been investigated deeply.

For the purpose to evaluate the integral effects of AWFT and various basalt fibre parameters on fresh behaviours of BFRM, 24 extra mortar groups with designated fibre dosages and fibre lengths were made and tested in this study, and a total of 68 mortar groups were used to carry out numerical analysis. Meanwhile, the parameters of fibre volume and fibre dimensions were combined to form a FF. Lastly, a multi-variable regression analysis was undertaken to reveal the roles of AWFT and FF in the fresh behaviours of BFRM.

## 2. Testing Program

### 2.1. Materials

Water, cement, sand, superplasticizer, and basalt fibres were used as the raw materials. For the water, local tap water was employed. For cement, a strength class 42.5R ordinary Portland cement (OPC) in conformance with Chinese Standard GB 175 [29] was employed. For sand, a local river sand was applied. Regarding the superplasticizer (SP), a polycarboxylate-based aqueous admixture was adopted. The basic properties of these raw materials are tabulated in Table 1.

**Figure 1:**
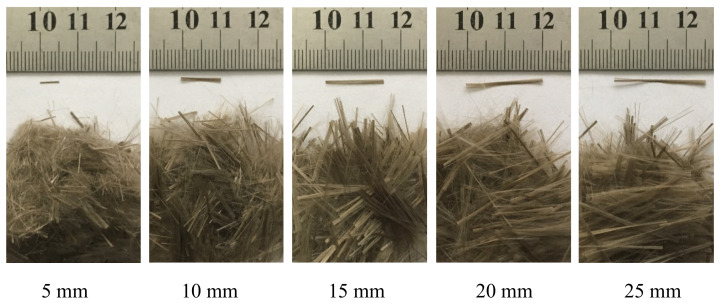
Photography of basalt fibres with different lengths.

Regarding the basalt fibres (BFs), they were imported from Ukrainian source. The fibres had a diameter of about 16 μm and different lengths of 5, 10, 15, 20, or 25 mm (as depicted in Figure 1). Other details of the fibres are listed in Table 2.

**Figure 2:**
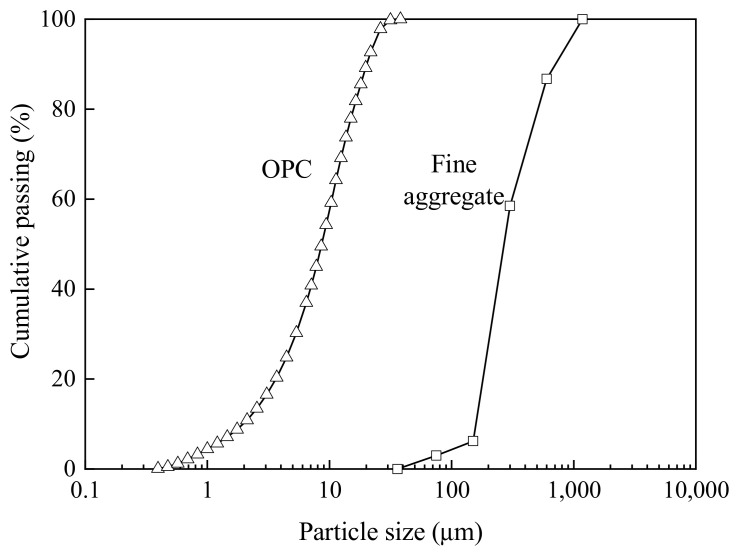
Particle size distributions of OPC and sand.

By using a laser diffraction particle size analyser (Mastersizer 3000, Malvern Panalytical Ltd., Malvern, UK), the particle size distribution (PSD) of the OPC was determined, and by means of mechanical sieving, the PSD of the fine aggregate was also determined. Their PSDs are plotted in Figure 2. From the PSDs, their specific surface areas (SSAs) can be calculated, as shown in Table 1. From the dimensions of the BF, the SSA of the BF was calculated, as given in Table 2.

### 2.2. Mix Design

In the previous study [27], in which the combined effects of AWFT and BF dosage were investigated, the BF dosage (expressed as a percentage of OPC content by mass) was set at 0.0%, 0.1%, 0.2%, 0.3%, 0.4%, and 0.5%, whereas the fibre length was fixed at 10 mm. In another study [28], in which the combined effects of AWFT and BF length were investigated, the BF dosage was fixed at 0.3%, whereas the fibre length was set at 5, 10, 15, 20, and 25 mm. In these two studies, the water to cement (W/C) ratio was designed as 0.25, 0.30, 0.35, and 0.40 by mass; the aggregate to cement (A/C) ratio was fixed at 1.00 by mass, and the SP dosage (expressed as percentage of the OPC content by mass) was given as 0.8%. The variations of the mix parameters of these two studies are listed in Table 3 and Table 4.

Herein, for the purpose of acquiring addition data to evaluate the integral effects of AWFT and BF parameters, a third testing plan was carried out. In this testing plan, the BF dosage was given at 0.2% and 0.4%, the fibre length was set at 5, 15, and 25 mm, and the W/C ratios applied were 0.25, 0.30, 0.35, and 0.40. Additionally, constant A/C ratio of 1.00 and SP dosage of 0.8% were adopted. The variation of the mix parameters of this research are listed in Table 5.

Overall, a total of 68 mortar groups were made for experimental evaluation. A sample code in the form of X–Y–Z was assigned to each mortar group: X denotes the fibre dosage; Y means the fibre length, and Z represents the W/C ratio, as listed in the left-most columns of Table 3, Table 4, Table 5, Table 6, Table 7 and Table 8.

### 2.3. Experimental Methods for Fresh Behaviours

The workability of the BFRM under static condition was assessed by applying a mini slump cone test [30], wherein the slump (the reduction in height of mortar patty) and the flow spread (the average diameter of mortar patty minus the base diameter of mini slump cone) were the measures of workability. The fluidity of the BFRM under dynamic condition was assessed by using a mini V-funnel test [30], in which the flow rate (the volume of V-funnel containing the mortar sample divided by the flow time) was the measure of fluidity. To interpret the test results, the larger the slump, flow spread, or flow rate values, the higher the workability or fluidity, and vice versa.

The cohesiveness of the BFRM was determined by employing a 1.18 mm sieve segregation test [30]. During the test, fresh mortar was poured to the sieve. The mass ratio of the mortar penetrated through the sieve and collected at the base receiver to the mass of mortar initially poured was defined as sieve segregation index (SSI). A larger SSI result means a lower cohesiveness, and vice versa.

The adhesiveness of the BFRM was evaluated by using a stone rod adhesion test [30]. In this test, stone rods were inserted and pulled out from the fresh mortar to determine the adhesion, defined as the mass of mortar adhered onto the stone rods divided by the surface area of the immersed parts of stone rods in the mortar. A larger adhesion value means a higher adhesiveness, and vice versa.

### 2.4. Experimental Method for Packing Density

In this program, the wet packing test developed by the authors’ research group [31,32,33,34] was adopted to determine the packing density of the solid component of the BFRM (OPC + sand + basalt fibre). In this test, the solid component was mixed with different amounts of water to form water–solid mixtures, and the respective solid-containing content of each water–solid mixture was measured. Among the solid-containing contents, the maximum solid-containing content was regarded as the wet packing density of the solid component of the BFRM. Detailed procedures of this test can be found in the related literature [35].

### 2.5. Determination of AWFT

The AWFT, having the physical meaning of the average thickness of water film coating the solid components [7,36,37], can be determined by the equation: AWFT = *u*_w_*’*/*A*_S_.

In this equation, *u*_w_*’* is the excess water ratio, which can be determined as: *u*_w_*’* = *u*_w_ − *u*_min_ (*u*_w_ is the volumetric ratio of water to all solid components, and *u*_min_ is the minimum voids ratio). As the packing density (*ϕ*_max_) of each BFRM group is measured, the minimum voids ratio can be calculated from the equation: *u*_min_ = (1 − *ϕ*_max_)/*ϕ*_max_.

In addition, *A*_S_ is the SSA of all the solid components. It can be calculated as: *A*_S_ = *A*_BF_ × *R*_BF_ + *A*_FA_ × *R*_FA_ + *A*_C_ × *R*_C_, where *A*_BF_, *A*_FA_, and *A*_C_ are the individual SSAs of basalt fibres, fine aggregate, and cement, respectively, and *R*_BF_, *R*_FA_, and *R*_C_ are the individual volumetric ratios of basalt fibres, fine aggregate, and cement to the total solid components, respectively.

### 2.6. Determination of Fibre Factor

To evaluate the integral effects of the fibre volume (fibre dosage by volume) and the fibre dimensions (BF length and diameter), the notion of fibre factor was brought in the previous studies [25,26,38]. After repeated trials, the following fibre factor was adopted in this study:

FF = V(L/D)(1)

where FF is fibre factor; V is the BF volume; L is the BF length; D is the BF diameter, and L/D is the aspect ratio. This FF is dimensionless and is relatively simple to comprehend.

## 3. Research Results

### 3.1. Results of Fresh Behaviours

The measured values of the fresh behaviours for the three experimental programs are listed in Table 3, Table 4 and Table 5. From the tables, the slump results varied from 5 mm to 55 mm; the flow spread varied from 0 mm to 213 mm; the flow rate varied from 0 mL/s to 594 mL/s; the SSI varied between 0.0% and 13.7%, and the adhesion varied between 0.051 g/cm^2^ and 0.175 g/cm^2^.

Regardless of the BF dosage and length, the slump, flow spread, and flow rate increased as the W/C ratio increased, meaning that the W/C ratio had positive effects on the workability and fluidity of the BFRM. The SSI was very low when the W/C ratio was at 0.25 or 0.30, but began to ascend to a higher W/C ratio, evidencing the negative influence of the W/C ratio on the cohesiveness of the BFRM. Furthermore, the adhesion first increased with the W/C ratio, but declined when the W/C ratio increased further. The turning point appeared at a W/C ratio of 0.30 to 0.35. Hence, the positive/negative influences of the W/C ratio on the adhesiveness rely on the corresponding W/C ratio.

However, apart from the W/C ratio, the BF dosage and length exhibited important roles in the fresh behaviours. From the tabulated values, at a given W/C ratio and fibre length, as the fibre dosage increased, all the test results of the fresh behaviours decreased, showing that the fibre dosage has adverse influences on the workability, fluidity, and adhesiveness but beneficial influence on the cohesiveness. In addition, at a fixed W/C ratio and fibre dosage, in general, a longer BF length resulted in a lower slump, flow spread, flow rate, SSI, and adhesion, indicating that the fibre length has adverse influences on the workability, fluidity, and adhesiveness but has beneficial influence on the cohesiveness.

### 3.2. Results of Packing Density

As shown in the second columns of Table 6, Table 7 and Table 8, the wet packing density of the solid component of the mortar groups varied from 0.7322 to 0.7458. Furthermore, the test results reveal that the BF dosage and length have an important impact on the packing density, as explained below.

From Table 6, it is apparent that when the fibre dosage increased from 0.0% to 0.2%, the corresponding packing density increased from 0.7358 to a peak value of 0.7453, but subsequently decreased as the fibre dosage further increased. This situation is reasonable and could be interpreted as follows: at a low fibre dosage, the fibres could be accommodated by the voids between the fine solid particles to densify the packing [39,40], whereas at a high fibre dosage, a certain amount of isolated fibres would be entrapped within the narrow gaps between the fine solid particles to wedge apart the solid particles (this is called the wedging effect), thus decreasing the packing density [41,42].

Table 7 shows that when increasing the fibre length from 5 mm to 25 mm, the corresponding packing density decreased from 0.7443 to 0.7343. Such a phenomenon may also be explicated by the wedging effect [41,42]: although the fibres were small in the radius and were rather flexible to readily penetrate into the voids between the fine solid particles, a certain amount of isolated fibres would be entrapped within the narrow gaps between the fine solid particles, especially when the fibres were relatively long. Consequentially, the fine solid particles were wedged apart so that the inter-particle distances increased and the packing density decreased.

### 3.3. Results of AWFT

The results of the water ratio and excess water ratio are given in the third and fourth columns of Table 6, Table 7 and Table 8, respectively. As expected, both the water ratio and the excess water ratio increased with the W/C ratio due to the presence of a higher water content. From the tables, some negative values of excess water ratio were found. The negative values mean that the water present was insufficient to fully occupy the voids, thus leaving some voids unfilled with water and causing entrapment of air in the voids within the solid skeleton.

Obviously, at a given volume of excess water, the larger the solid surface area, the thinner the water films. From the solid proportions and particle size distributions, the SSAs were calculated and are listed in the fifth columns of Table 6, Table 7 and Table 8. From these tabulated values, the SSA of the mortar groups varied between 715,010 m^2^/m^3^ and 716,307 m^2^/m^3^.

**Figure 3:**
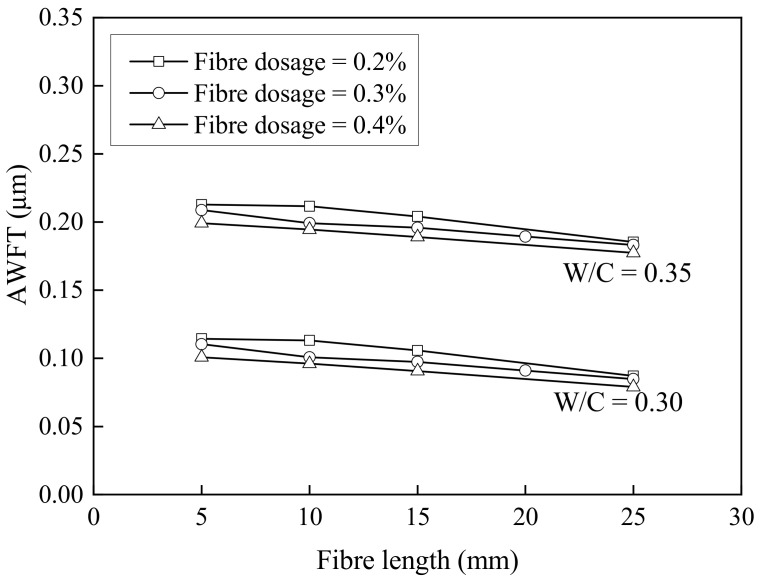
AWFT versus basalt fibre length for different fibre dosages.

Lastly, the AWFT values were calculated and are presented in the sixth columns of Table 6, Table 7 and Table 8. The AWFT ranged from −0.0193 μm to 0.3113 μm. To illustrate how the AWFT changed with the W/C ratio, the typical BF dosage and BF length results are plotted in Figure 3. It is obvious that the AWFT increased with the W/C ratio. Regardless of the W/C ratio, generally, the increase in the BF dosage and/or BF length would reduce the AWFT; this is in line with the decrease in packing density due to the addition of fibres.

**Figure 4:**
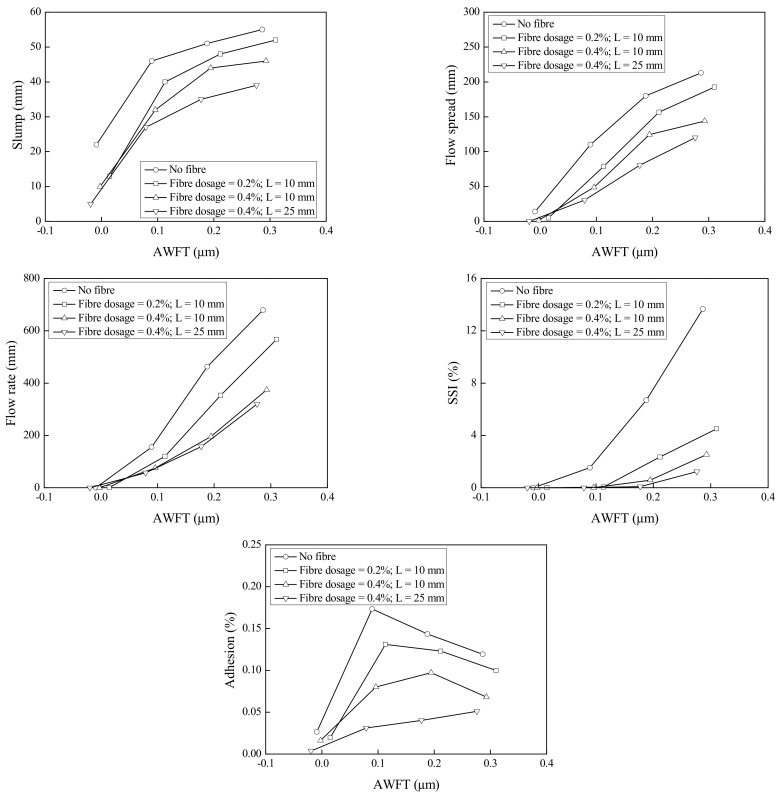
Fresh behaviours of BFRM versus AWFT for different fibre dosages and lengths.

To illustrate how the AWFT and fibres affect the fresh behaviours, the test results of some typical mortar groups are plotted in Figure 4. It is evident that, in general, as the AWFT increased, the slump, flow spread, flow rate, and SSI gradually increased, and the adhesion first increased to a peak and then decreased. It means that the AWFT plays a significant role in the fresh behaviours of BFRM. Furthermore, it is evident that the increases in fibre dosage and/or fibre length would shift the curves of the fresh properties downwards, indicating that the BFs also have important influences on the fresh behaviours.

### 3.4. Results of Fibre Factor

To investigate the integral effects of the fibre dosage and fibre dimensional parameters, the fibre factor (FF), defined as the fibre volume V times the fibre aspect ratio L/D, was determined. It is obvious that the value of FF increases with the fibre volume and the fibre length. In the following, the fibre volume was converted from the fibre dosage by mass. After the conversion, the results of the fibre volume were tabulated in the seventh columns of Table 6, Table 7 and Table 8.

Having obtained the fibre volume and with reference to the dimensions of the fibre, the corresponding FF can be evaluated by using Equation (1). The resulting FF values are shown in the right-most columns of Table 6, Table 7 and Table 8, where it can be found that the FF values range from 0.0000 to 2.5719. As expected, the FF increased as the fibre volume and/or the fibre length increased.

## 4. Integral Influences of AWFT and FF

### 4.1. Effects on Workability

**Figure 5:**
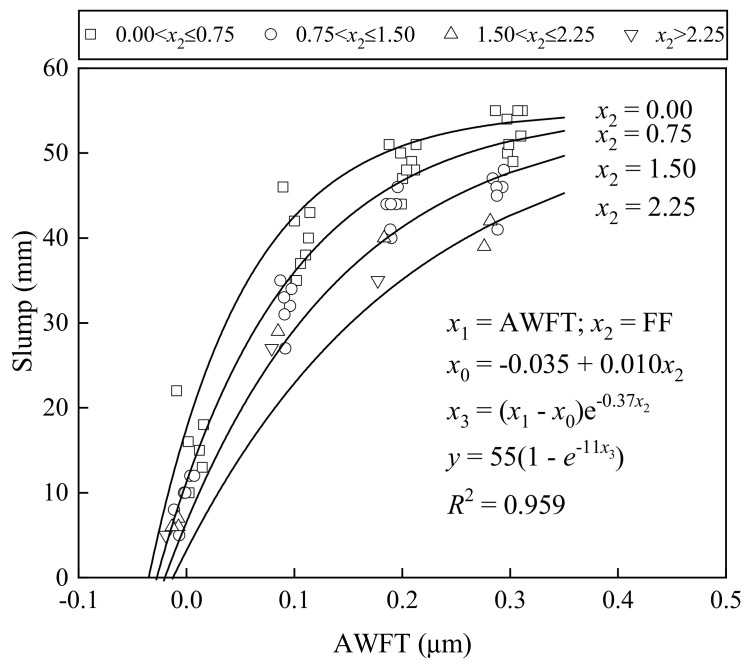
Correlation of slump with AWFT and FF.

**Figure 6:**
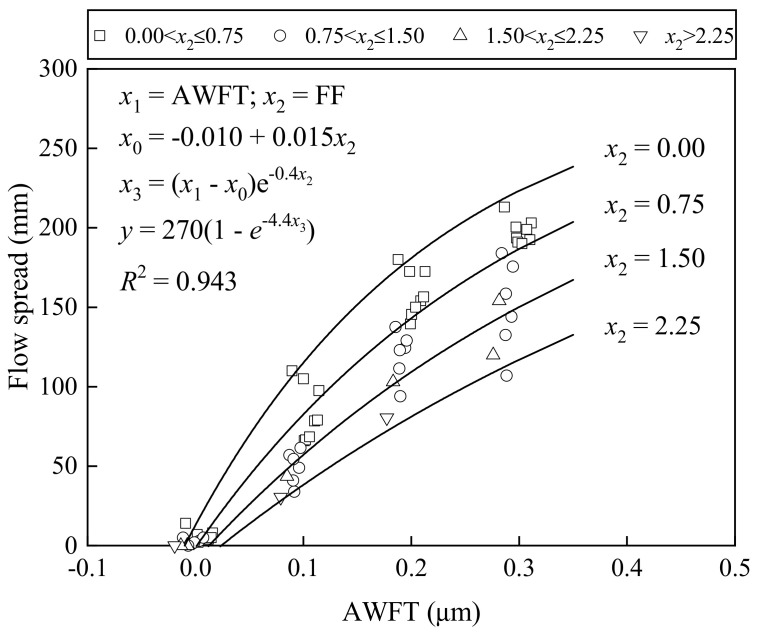
Correlation of flow spread with AWFT and FF.

Figure 5 shows how the slump varied with the AWFT at different FFs, whereas Figure 6 presents how the flow spread changed with the AWFT at different FFs. These figures show that both the slump and flow spread increased with the AWFT at a gradually decreasing rate. Basically, the AWFT is a major and positive parameter influencing the workability of BFRM. Moreover, at a fixed AWFT, a larger FF always led to a smaller slump and flow spread. Therefore, the FF has a direct but negative influence on the workability.

To evaluate the integral influence of the AWFT and FF on the workability of BFRM, a multi-variable regression analysis was applied to reveal the slump–AWFT as well as the flow spread–AWFT correlation at various FFs. The best-fit curves and the corresponding formulas are listed in the figures for reference. It should be noted that the corresponding coefficient of determination, or *R*^2^ values (0.959 and 0.940), were very high, demonstrating that the workability of BFRM is mainly governed by the two parameters: AWFT and FF.

### 4.2. Effects on Fluidity

**Figure 7:**
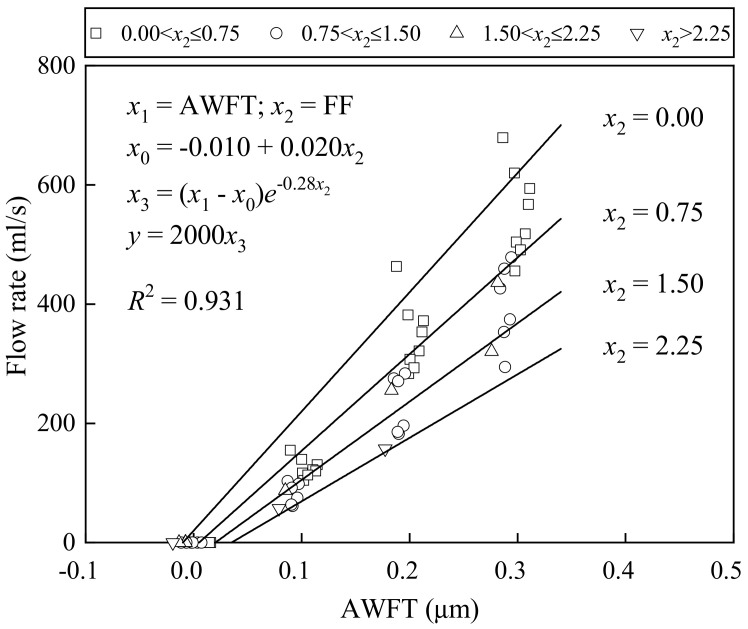
Correlation of flow rate with AWFT and FF.

Figure 7 presents how the flow rate changed with the AWFT at different FFs: when the AWFT was around zero or negative, there was no flow rate, and as the AWFT further increased, the flow rate increased approximately linearly with the AWFT. This phenomenon shows that the AWFT should be a dominant and positive parameter influencing the fluidity of BFRM. In addition, the test results reflect that regardless of the AWFT, the flow rate became lower as the FF became higher, suggesting that FF plays a direct but negative role in the fluidity.

To quantify the integral effects of the AWFT and FF on the fluidity of BFRM, a multi-variable regression analysis was launched. The derived formulas and the best-fit curves for the flow rate–AWFT relations at different FFs are depicted in the Figure 7. The *R*^2^ value obtained was 0.931, which is considerably high, showing that the fluidity of BFRM is mainly governed by the AWFT and FF.

### 4.3. Effects on Cohesiveness

**Figure 8:**
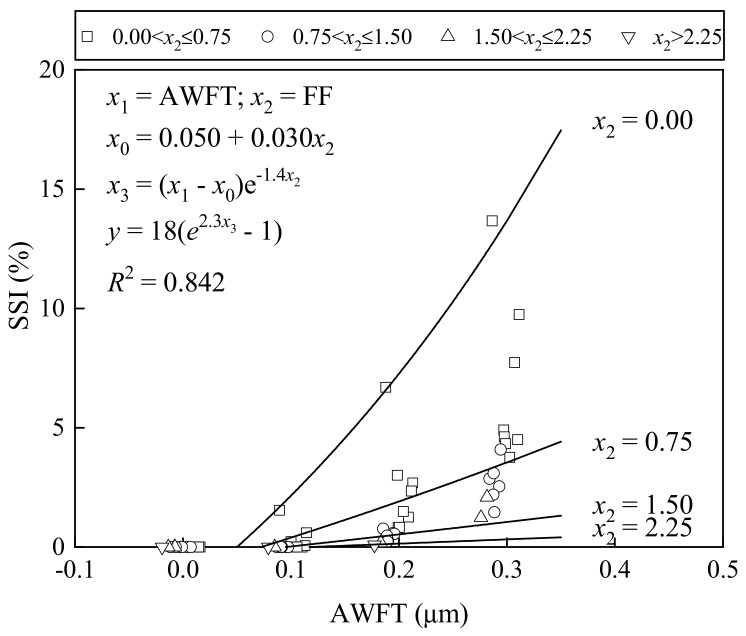
Correlation of SSI with AWFT and FF.

To show how the FF and AWFT influence the cohesiveness of BFRM, the SSI values are plotted versus the AWFT for different FFs in Figure 8. It was found that when the AWFT was close to zero, the SSI was equal to zero. As the AWFT increased to about 0.1 μm, the SSI increased at a gradually increasing rate. Hence, the effect of the AWFT on the cohesiveness of BFRM should be major but negative. On the other hand, it is also noted that at a given AWFT, the increase in FF would greatly reduce the SSI, suggesting that the FF plays a major beneficial role in the cohesiveness.

For the purpose of studying the integral influence of the AWFT and FF, the formulas for the SSI–AWFT relation at different FFs were derived by performing a regression analysis as presented in Figure 8. The fitted curves are also shown in the figure. The *R*^2^ value obtained was 0.842, which is reasonably good, meaning that both the AWFT and FF are governing parameters for the cohesiveness of BFRM.

### 4.4. Effects on Adhesiveness

**Figure 9:**
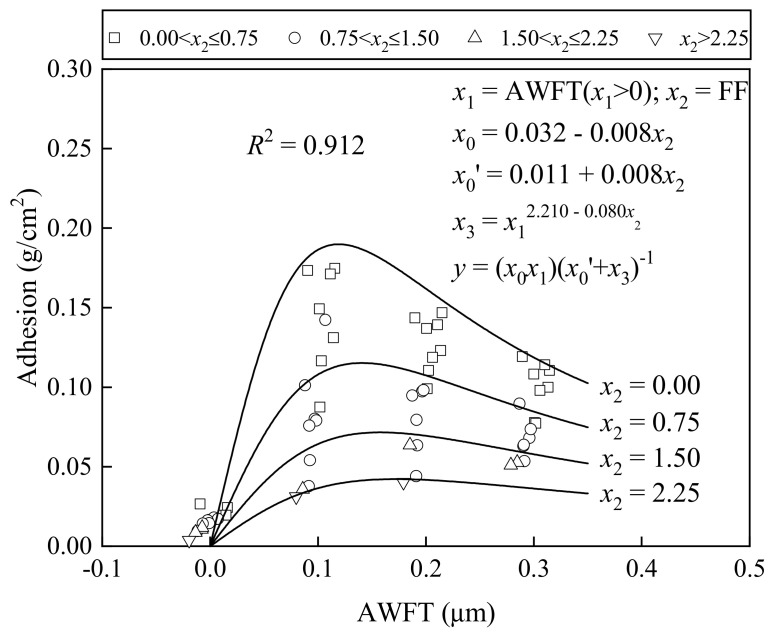
Correlation of adhesion with AWFT and FF.

Figure 9 exhibits how the adhesion varied with the AWFT at different FFs: when the AWFT was close to zero, the adhesion was rather low; as the AWFT ascended to around 0.10 to 0.20 μm, the adhesion reached a peak value dependent on the FF, but beyond the peak, the adhesion reduced as the AWFT increased further. This means that the AWFT has a beneficial/adverse influence on the adhesiveness. Furthermore, given the same AWFT, the adhesion also varied with the FF. In general, the increase in FF significantly reduced the adhesion.

The AWFT and FF were applied as variables in a multi-variable regression analysis to assess their integral effects on the adhesiveness of the BFRM. The resulting fitting curves and formulas are exhibited in the figure. A high *R*^2^ value of 0.912 was yielded, indicating that the adhesiveness of the BFRM is mainly governed by both the AWFT and FF.

## 5. Discussions

Summing up, the analysis of the experimental results and the correlations of the slump, flow spread, flow rate, SSI, and adhesion with the AWFT and FF reveal that the concept of AWFT is also applicable to BFRM in the sense that AWFT is also a key parameter governing the fresh behaviours of BFRM and that the effects of the BF added may be accounted for by a single FF defined as V (L/D) whereby the influences of the fibre volume and aspect ratio are included. All in all, both the AWFT and FF play important roles in the fresh behaviours of BFRM.

Whilst the influence of the AWFT on the fresh behaviour of BFRM is obvious because the AWFT functions as a lubricating layer in the water–solid mixture, the influence of the fibres added is more complicated. Basically, the fibres added exert their influences in two distinct ways. First, the fibres indirectly influence the AWFT by increasing/decreasing the packing density and changing the SSA of the solid component of BFRM. Second, the fibres directly influence the FF, which generally possesses negative impacts on the workability, fluidity, and adhesiveness and positive impacts on the cohesiveness. These two distinct influences together render the net influences of the various fibre parameters rather complicated and difficult to model.

Nevertheless, the rather high *R*^2^ values obtained in the regression analysis indicate that the AWFT and FF together already govern the fresh behaviours of BFRM and that these two parameters may be used to develop a theoretical model for the fresh properties of BFRM. In this regard, further research is highly recommended. Apart from improving our understanding of the fresh behaviours of BFRM, the development of a theoretical model has high practical values for mortar production. With the availability of a theoretical model for the fresh properties, the mix design of BFRM may be preliminary optimised for given targeted fresh properties before conducting cumbersome laboratory and plant trials. This could partially relieve the time, manpower, and materials required for conducting trials.

However, it is worth noting that the actual effects of the FF are also dependent on the types of fibres used. Generally, rigid fibres (as exemplified by steel fibres) and flexible fibres (as exemplified by polypropylene and basalt fibres) affect the fresh behaviours of FRCBM in different manners. In addition, among the flexible fibres, fibres with a different elastic modulus may also have somewhat different effects. Hence, the correlation formulas presented herein for predicting the fresh behaviours are only applicable to BFRM. For different types of fibres, separate studies are needed to establish the correlation formulas specifically for the types of fibres to be used.

## 6. Conclusions

A total of three research programs were completed to generate sufficient test results for analysing the integral effects of the average water film thickness (AWFT), basalt fibres (BF) dosage, and length on fresh behaviours of basalt fibre-reinforced mortar (BFRM) and for the possible establishment of a single fibre factor (FF) to consider the direct influences of the fibres. Putting all the test results from these programs together for analysis and correlating the test results to both the AWFT and FF, some conclusions could be drawn as follows:

1. The concept of AWFT, which has been proven to be applicable to plain mortars without fibres added, is also applicable to BFRM in the sense that the AWFT is a key parameter governing the fresh behaviours of BFRM;
2. Basically, the AWFT possesses positive influences on the workability and fluidity, negative impacts on the cohesiveness, and positive or negative effects on the adhesiveness depending on the AWFT;
3. Aside from the AWFT, both the fibre dosage and length have negative influences on the workability, fluidity, and adhesiveness but positive effects on the cohesiveness of BFRM;
4. Apart from the above direct effects, the fibre dosage has a positive or negative impact on the packing density depending on the fibre dosage, whereas the fibre length always has a negative impact on the packing density. In consideration of the slight alteration of the specific surface area in the solid component of BFRM, the fibres added exerted certain indirect effects on the AWFT;
5. The combined effects of the fibre dosage and the aspect ratio could be accounted for by a single FF defined as V (L/D). This FF is dimensionless and is relatively simple to comprehend;
6. Correlations of the slump, flow spread, flow rate, SSI, and adhesion with both the AWFT and FF by means of a multi-variable regression analysis yielded a rather high coefficient of determination or the *R*^2^ values, suggesting that the AWFT and FF are together the key parameters governing the fresh behaviours of BFRM;
7. Hence, the two parameters AWFT and FF may be used to develop a model for the fresh properties of BFRM, which could have a high theoretical value for improving our understanding of the fresh behaviours of BFRM, as well as a high practical value for enabling a preliminary mix design optimization before conducting cumbersome laboratory and plant trials.

## Figures and Tables

**Table 1:** Properties of raw materials.

Properties	Raw Materials			
	Water	Cement	Fine Aggregate	Super- Plasticizer
Specific gravity	1.00	3.11	2.66	1.08
Moisture content	-	-	0.04%	-
Water absorption	-	-	1.10%	-
Max. particle size (mm)	-	-	1.18	-
Solid content	-	-	-	20%
Specific surface area (m^2^/m^3^)	-	1.95 × 10^6^	3.16 × 10^4^	-

**Table 2:** Properties of basalt fibres.

Fibre Type	Specific Gravity	Diameter (μm)	Elastic Modulus (GPa)	Tensile Strength (MPa)	Elongation at Break (%)	Specific Surface Area (m^2^/m^3^)
Basalt fibre	2.53	16 ± 2	84	1450	2.8	2.50 × 10^5^

**Table 3:** Test results of mortar samples with varying fibre dosage and length = 10 mm.

Sample Code	Slump (mm)	Flow Spread (mm)	Flow Rate (mL/s)	SSI (%)	Adhesion (g/cm^2^)
0.0%-0-0.25	22	14	0	0.0	0.027
0.0%-0-0.30	46	110	155	1.5	0.173
0.0%-0-0.35	51	180	463	6.7	0.144
0.0%-0-0.40	55	213	679	13.7	0.119
0.1%-10-0.25	16	7	0	0.0	0.017
0.1%-10-0.30	42	105	139	0.2	0.149
0.1%-10-0.35	50	173	382	3.0	0.137
0.1%-10-0.40	54	201	620	4.9	0.108
0.2%-10-0.25	13	5	0	0.0	0.019
0.2%-10-0.30	40	79	120	0.1	0.131
0.2%-10-0.35	48	157	353	2.3	0.123
0.2%-10-0.40	52	193	567	4.5	0.100
0.3%-10-0.25	12	3	0	0.0	0.018
0.3%-10-0.30	35	67	104	0.0	0.117
0.3%-10-0.35	47	146	307	0.8	0.110
0.3%-10-0.40	51	191	504	4.3	0.077
0.4%-10-0.25	10	2	0	0.0	0.016
0.4%-10-0.30	32	49	75	0.0	0.080
0.4%-10-0.35	44	125	196	0.6	0.097
0.4%-10-0.40	46	144	374	2.5	0.068
0.5%-10-0.25	5	0	0	0.0	0.014
0.5%-10-0.30	27	34	61	0.0	0.054
0.5%-10-0.35	40	94	182	0.3	0.063
0.5%-10-0.40	41	107	295	1.5	0.054

**Table 4:** Test results of mortar samples with fibre dosage = 0.3% and varying length.

Sample Code	Slump (mm)	Flow Spread (mm)	Flow Rate (mL/s)	SSI (%)	Adhesion (g/cm^2^)
0.3%-5-0.25	15	4	0	0.0	0.019
0.3%-5-0.30	38	79	122	0.0	0.171
0.3%-5-0.35	49	154	321	1.2	0.139
0.3%-5-0.40	55	199	518	7.7	0.114
0.3%-10-0.25	12	3	0	0.0	0.018
0.3%-10-0.30	35	67	104	0.0	0.117
0.3%-10-0.35	47	146	307	0.8	0.110
0.3%-10-0.40	51	191	504	4.3	0.077
0.3%-15-0.25	10	2	0	0.0	0.014
0.3%-15-0.30	34	62	98	0.0	0.079
0.3%-15-0.35	46	129	284	0.6	0.098
0.3%-15-0.40	48	176	478	4.1	0.074
0.3%-20-0.25	6	0	0	0.0	0.012
0.3%-20-0.30	31	55	92	0.0	0.076
0.3%-20-0.35	44	123	271	0.2	0.079
0.3%-20-0.40	45	159	459	3.1	0.064
0.3%-25-0.25	6	0	0	0.0	0.009
0.3%-25-0.30	29	44	87	0.0	0.036
0.3%-25-0.35	40	103	255	0.2	0.064
0.3%-25-0.40	42	154	436	2.1	0.053

**Table 5:** Test results of mortar samples with varying fibre dosage and varying length.

Sample Code	Slump (mm)	Flow Spread (mm)	Flow Rate (mL/s)	SSI (%)	Adhesion (g/cm^2^)
0.2%-5-0.25	18	8	0	0.0	0.024
0.2%-5-0.30	43	98	130	0.6	0.175
0.2%-5-0.35	51	173	372	2.7	0.147
0.2%-5-0.40	55	203	594	9.7	0.110
0.2%-15-0.25	12	5	0	0.0	0.017
0.2%-15-0.30	37	69	113	0.0	0.142
0.2%-15-0.35	48	150	293	1.5	0.119
0.2%-15-0.40	49	190	491	3.8	0.098
0.2%-25-0.25	8	5	0	0.0	0.010
0.2%-25-0.30	35	57	103	0.0	0.101
0.2%-25-0.35	44	138	275	0.8	0.095
0.2%-25-0.40	47	184	426	2.9	0.090
0.4%-5-0.25	10	2	0	0.0	0.017
0.4%-5-0.30	35	66	117	0.0	0.088
0.4%-5-0.35	44	140	284	0.8	0.099
0.4%-5-0.40	50	194	455	4.6	0.078
0.4%-15-0.25	7	0	0	0.0	0.011
0.4%-15-0.30	33	41	64	0.0	0.038
0.4%-15-0.35	41	112	186	0.5	0.044
0.4%-15-0.40	46	133	353	2.2	0.063
0.4%-25-0.25	5	0	0	0.0	0.004
0.4%-25-0.30	27	31	57	0.0	0.031
0.4%-25-0.35	35	81	157	0.1	0.040
0.4%-25-0.40	39	120	320	1.2	0.051

**Table 6:** Packing density and AWFT of mortar samples with varying fibre dosage and length = 10 mm.

Sample Code	Packing Density	Water Ratio	Excess Water Ratio	Specific Surface Area (m^2^/m^3^)	AWFT (μm)	Fibre Volume (%)	Fibre Factor
0.0%-0-0.25	0.7358	0.3526	−0.0065	716,307	−0.0091	0.0000	0.0000
0.0%-0-0.30		0.4232	0.0641		0.0895	0.0000	0.0000
0.0%-0-0.35		0.4937	0.1346		0.1879	0.0000	0.0000
0.0%-0-0.40		0.5642	0.2051		0.2863	0.0000	0.0000
0.1%-10-0.25	0.7401	0.3524	0.0012	716,047	0.0017	0.0412	0.2575
0.1%-10-0.30		0.4229	0.0717		0.1001	0.0392	0.2448
0.1%-10-0.35		0.4934	0.1422		0.1986	0.0373	0.2332
0.1%-10-0.40		0.5639	0.2127		0.2970	0.0356	0.2227
0.2%-10-0.25	0.7453	0.3522	0.0105	715,787	0.0147	0.0824	0.5148
0.2%-10-0.30		0.4227	0.0810		0.1132	0.0783	0.4893
0.2%-10-0.35		0.4931	0.1514		0.2115	0.0746	0.4662
0.2%-10-0.40		0.5636	0.2219		0.3100	0.0712	0.4452
0.3%-10-0.25	0.7410	0.3521	0.0026	715,528	0.0036	0.1235	0.7719
0.3%-10-0.30		0.4225	0.0730		0.1020	0.1174	0.7337
0.3%-10-0.35		0.4929	0.1434		0.2004	0.1119	0.6991
0.3%-10-0.40		0.5633	0.2138		0.2988	0.1068	0.6676
0.4%-10-0.25	0.7388	0.3519	−0.0016	715,269	−0.0022	0.1646	1.0288
0.4%-10-0.30		0.4222	0.0687		0.0960	0.1565	0.9779
0.4%-10-0.35		0.4926	0.1391		0.1945	0.1491	0.9318
0.4%-10-0.40		0.5630	0.2095		0.2929	0.1424	0.8898
0.5%-10-0.25	0.7372	0.3517	−0.0048	715,010	−0.0067	0.2057	1.2854
0.5%-10-0.30		0.4220	0.0655		0.0916	0.1955	1.2218
0.5%-10-0.35		0.4923	0.1358		0.1899	0.1863	1.1643
0.5%-10-0.40		0.5627	0.2062		0.2884	0.1779	1.1119

**Table 7:** Packing density and AWFT with fibre dosage = 0.30% and varying length.

Sample Code	Packing Density	Water Ratio	Excess Water Ratio	Specific Surface Area (m^2^/m^3^)	AWFT (μm)	Fibre Volume (%)	Fibre Factor
0.3%-5-0.25	0.7443	0.3521	0.0086	715,528	0.0120	0.1235	0.3859
0.3%-5-0.30		0.4225	0.0790		0.1104	0.1174	0.3668
0.3%-5-0.35		0.4929	0.1494		0.2088	0.1119	0.3495
0.3%-5-0.40		0.5633	0.2198		0.3072	0.1068	0.3338
0.3%-10-0.25	0.7405	0.3521	0.0017	715,528	0.0024	0.1235	0.7719
0.3%-10-0.30		0.4225	0.0721		0.1008	0.1174	0.7337
0.3%-10-0.35		0.4929	0.1425		0.1992	0.1119	0.6991
0.3%-10-0.40		0.5633	0.2129		0.2975	0.1068	0.6676
0.3%-15-0.25	0.7392	0.3521	−0.0007	715,528	−0.0010	0.1235	1.1578
0.3%-15-0.30		0.4225	0.0697		0.0974	0.1174	1.1005
0.3%-15-0.35		0.4929	0.1401		0.1958	0.1119	1.0486
0.3%-15-0.40		0.5633	0.2105		0.2942	0.1068	1.0014
0.3%-20-0.25	0.7367	0.3521	−0.0053	715,528	−0.0074	0.1235	1.5438
0.3%-20-0.30		0.4225	0.0651		0.0910	0.1174	1.4674
0.3%-20-0.35		0.4929	0.1355		0.1894	0.1119	1.3982
0.3%-20-0.40		0.5633	0.2059		0.2878	0.1068	1.3352
0.3%-25-0.25	0.7343	0.3521	−0.0097	715,528	−0.0136	0.1235	1.9297
0.3%-25-0.30		0.4225	0.0607		0.0848	0.1174	1.8342
0.3%-25-0.35		0.4929	0.1311		0.1832	0.1119	1.7477
0.3%-25-0.40		0.5633	0.2015		0.2816	0.1068	1.6690

**Table 8:** Packing density and AWFT of mortar samples with varying fibre dosage and varying length.

Sample Code	Packing Density	Water Ratio	Excess Water Ratio	Specific Surface Area (m^2^/m^3^)	AWFT (μm)	Fibre Volume (%)	Fibre Factor
0.2%-5-0.25	0.7458	0.3522	0.0114	715,787	0.0159	0.0824	0.2574
0.2%-5-0.30		0.4227	0.0819		0.1144	0.0783	0.2447
0.2%-5-0.35		0.4931	0.1523		0.2128	0.0746	0.2331
0.2%-5-0.40		0.5636	0.2228		0.3113	0.0712	0.2226
0.2%-15-0.25	0.7424	0.3522	0.0052	715,787	0.0073	0.0824	0.7722
0.2%-15-0.30		0.4227	0.0757		0.1058	0.0783	0.7340
0.2%-15-0.35		0.4931	0.1461		0.2041	0.0746	0.6993
0.2%-15-0.40		0.5636	0.2166		0.3026	0.0712	0.6678
0.2%-25-0.25	0.7351	0.3522	−0.0082	715,787	−0.0115	0.0824	1.2870
0.2%-25-0.30		0.4227	0.0623		0.0870	0.0783	1.2233
0.2%-25-0.35		0.4931	0.1327		0.1854	0.0746	1.1656
0.2%-25-0.40		0.5636	0.2032		0.2839	0.0712	1.1130
0.4%-5-0.25	0.7407	0.3519	0.0018	715,269	0.0025	0.1646	0.5144
0.4%-5-0.30		0.4222	0.0721		0.1008	0.1565	0.4889
0.4%-5-0.35		0.4926	0.1425		0.1992	0.1491	0.4659
0.4%-5-0.40		0.5630	0.2129		0.2977	0.1424	0.4449
0.4%-15-0.25	0.7370	0.3519	−0.0055	715,269	−0.0077	0.1646	1.5431
0.4%-15-0.30		0.4222	0.0648		0.0906	0.1565	1.4668
0.4%-15-0.35		0.4926	0.1352		0.1890	0.1491	1.3976
0.4%-15-0.40		0.5630	0.2056		0.2874	0.1424	1.3347
0.4%-25-0.25	0.7322	0.3519	−0.0138	715,269	−0.0193	0.1646	2.5719
0.4%-25-0.30		0.4222	0.0565		0.0790	0.1565	2.4447
0.4%-25-0.35		0.4926	0.1269		0.1774	0.1491	2.3294
0.4%-25-0.40		0.5630	0.1973		0.2758	0.1424	2.2245

## Data Availability

The data presented in this study are available on request from the corresponding author.
